# Changes in tuberculosis risk after transplantation in the setting of decreased community tuberculosis incidence: a national population-based study, 2008–2020

**DOI:** 10.1186/s12941-023-00661-4

**Published:** 2024-01-03

**Authors:** JongHoon Hyun, Myeongjee Lee, Inkyung Jung, Eunhwa Kim, Seung Min Hahn, Yu Ri Kim, Sungmin Lim, Kyong Ihn, Min Young Kim, Jong Gyun Ahn, Joon-Sup Yeom, Su Jin Jeong, Ji-Man Kang

**Affiliations:** 1 Division of Infectious Diseases, Department of Internal Medicine, Inje University Ilsan Paik Hospital, Goyang, Republic of Korea; 2 Department of Internal Medicine, Yonsei University College of Medicine, Seoul, South Korea; 3 Department of Biomedical Systems Informatics, Biostatistics Collaboration Unit, Yonsei University College of Medicine, Seoul, Republic of Korea; 4 Division of Biostatistics, Department of Biomedical Systems Informatics, Yonsei University College of Medicine, Seoul, Republic of Korea; 5 Department of Pediatric Hematology-Oncology, Yonsei Cancer Center, Yonsei University College of Medicine, Seoul, Republic of Korea; 6 Division of Hematology, Department of Internal Medicine, Severance Hospital, Yonsei University College of Medicine, Seoul, Republic of Korea; 7 Department of Pediatrics, Severance Children’s Hospital, Yonsei University College of Medicine, 50-1 Yonsei-Ro, Seodaemun-Gu, Seoul, 03722 Republic of Korea; 8 Department of Pediatric Surgery, Department of Surgery, Severance Children’s Hospital, Yonsei University College of Medicine, Seoul, Republic of Korea; 9 Institute for Immunology and Immunological Diseases, Yonsei University College of Medicine, Seoul, Republic of Korea; 10 Division of Infectious Disease, Department of Internal Medicine, Severance Hospital, Yonsei University College of Medicine, 50-1 Yonsei-Ro Seodaemun-Gu, Seoul, 03722 Republic of Korea

**Keywords:** Solid organ transplantation, Hematopoietic stem cell transplantation, Tuberculosis, Community tuberculosis burden, Standardized incidence ratio

## Abstract

**Background:**

Transplant recipients are immunocompromised and vulnerable to developing tuberculosis. However, active tuberculosis incidence is rapidly declining in South Korea, but the trend of tuberculosis infection among transplant recipients has not been elucidated. This study aimed to evaluate the risk of active tuberculosis after transplantation, including risk factors for tuberculosis and standardized incidence ratios, compared with that in the general population.

**Methods:**

This retrospective study was conducted based on the South Korean health insurance review and assessment database among those who underwent transplantation (62,484 recipients) between 2008 and 2020. Tuberculosis incidence was compared in recipients treated during higher- (2010–2012) and lower-disease burden (2016–2018) periods. Standardized incidence ratios were analyzed using the Korean Tuberculosis Surveillance System. The primary outcome was the number of new tuberculosis cases after transplantation.

**Results:**

Of 57,103 recipients analyzed, the overall cumulative incidence rate 1 year after transplantation was 0.8% (95% confidence interval [CI]: 0.7–0.8), significantly higher in the higher-burden period than in the lower-burden period (1.7% vs. 1.0% 3 years after transplantation, *P* < 0.001). Individuals who underwent allogeneic hematopoietic stem cell transplantation had the highest tuberculosis incidence, followed by those who underwent solid organ transplantation and autologous hematopoietic stem cell transplantation (*P* < 0.001). The overall standardized incidence ratio was 3.9 (95% CI 3.7–4.2) and was the highest in children aged 0–19 years, at 9.0 (95% CI 5.7–13.5). Male sex, older age, tuberculosis history, liver transplantation, and allogeneic hematopoietic stem cell transplantation were risk factors for tuberculosis.

**Conclusions:**

Transplant recipients are vulnerable to developing tuberculosis, possibly influenced by their immunocompromised status, solid organ transplant type, age, and community prevalence of tuberculosis. Tuberculosis prevalence by country, transplant type, and age should be considered to establish an appropriate tuberculosis prevention strategy for high-risk groups.

**Graphical Abstract:**

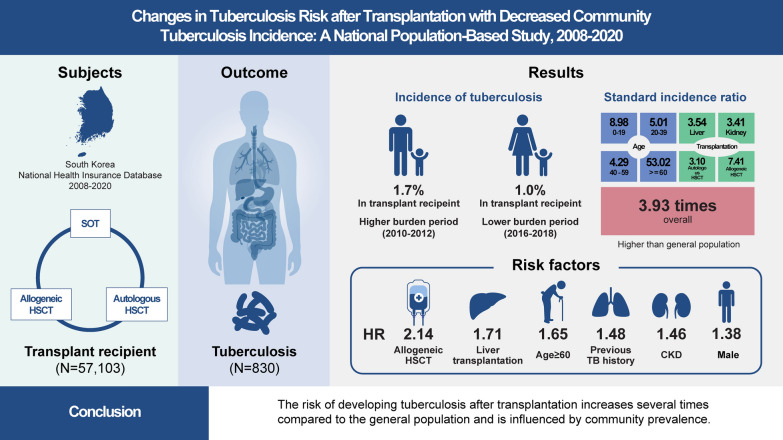

**Supplementary Information:**

The online version contains supplementary material available at 10.1186/s12941-023-00661-4.

## Background

Transplantation is a complex, multidisciplinary procedure that requires considerable medical resources and is used to treat various intractable diseases or end-stage organ failure. Recipients of transplants are administered immunosuppressive therapy, which exposes them to opportunistic infections such as tuberculosis. Tuberculosis remains a prevalent disease worldwide, with an estimated 10.6 million new active tuberculosis cases in 2021, making it the second leading cause of death among infectious diseases after coronavirus disease [1]. In the pathogenesis of tuberculosis, exogenous exposure to *Mycobacterium tuberculosis* and suppression or senescence of the host immunity are crucial endogenous factors [2, 3]. Therefore, transplant recipients in highly endemic areas, a representative immunocompromised population, are a high-risk group for opportunistic tuberculosis infection [4]. The incidence of tuberculosis in recipients of solid organ transplants (SOTs) varies from 1.2 to 6.4%, reaching 15% in highly endemic areas [5, 6]. Tuberculosis incidence in recipients of hematopoietic stem cell transplants (HSCTs) is 0.0014–16% [7]. However, the current data in the literature have been obtained from different countries with varied incidence rates of active tuberculosis. Thus, comparing such data is limited by detection bias due to differences in the actual tuberculosis surveillance systems across countries.

Tuberculosis was previously “endemic” in South Korea, according to the World Health Organization category (100–299 new and relapse cases per 100,000 population per year) [1]. Since 2011, active tuberculosis management and aggressive tracing and treatment of latent tuberculosis have been implemented through the nationwide public–private tuberculosis management project. Consequently, the number of new tuberculosis cases decreased sharply from 100.8 per 100,000 population per year in 2011 to 49.4 per 100,000 population per year in 2020 (Additional file 1: Table S1) [8]. Notably, thousands of transplants are performed yearly in South Korea (Additional file 2: Table S2), providing an excellent opportunity to observe differences in the incidence of active tuberculosis among transplant recipients in settings with different tuberculosis incidence rates using the same population-based database. This study aimed to evaluate the risk of active tuberculosis after transplantation, including the risk factors for active tuberculosis and standardized incidence ratios (SIRs), compared with that in the general population.

## Methods

### Study design and population

This was a population-based, retrospective cohort study. We used the nationwide claims database of the Health Insurance Review and Assessment (HIRA) in South Korea. The HIRA reviews all healthcare claims from the National Health Insurance Service (NHIS), a universal insurer covering 97% of the South Korean population [9]. The HIRA database contains the age and sex of the insured individual; healthcare provider type; diagnosis code based on the International Classification of Disease, Revision 10, Clinical Modification (ICD-10-CM); and procedures and prescriptions covered by the NHIS.

Transplant recipients were individuals with procedure codes for transplantation claimed between January 1, 2008, and December 31, 2020. The detailed procedure codes for transplantation are described in Additional file 3: Table S3. When the same procedure code persisted for HSCT within 60 days, it was considered a single transplantation case (tandem autologous HSCT). Simultaneous kidney and pancreatic transplantations were considered for kidney transplantation. Small intestine transplantation and pancreas transplantation alone were classified as other transplantation types. However, dual transplantations other than those mentioned above, re-SOT with an interval of ≥ 30 days, and corneal and scleral transplantations were not included in this study. Data from 2008 were excluded for washout. Recipients who died on the day of transplantation were excluded. Patients diagnosed with tuberculosis within the last year before transplantation were also excluded because diagnostic codes may persist after transplantation, making it difficult to distinguish between pre-existing and new infections.

### Definitions

As a primary outcome, tuberculosis was defined as active tuberculosis with pulmonary or extrapulmonary tuberculosis codes and one or more prescriptions for tuberculosis (Additional file 4: Table S4). When both codes were applicable, they were classified as pulmonary tuberculosis. Tuberculosis history was defined as the presence of diagnostic codes for active tuberculosis 1 year before transplantation. Comorbidity was defined as the presence of one or more of the diagnostic codes summarized in Additional file 5: Table S5 within a year of transplantation [10]. Death was defined as death at discharge with no subsequent claims. To compare the impact of tuberculosis burden in the general population on the timing of transplantation, burden periods were selected based on the incidence rate in the general population. The highest incidence rate (96.4–98.4 per 100,000 population per year) was observed in 2010–2012, and this was defined as the higher-burden period. In contrast, the lowest incidence rate (76.8–65.9 per 100,000 population per year) was observed in 2016–2018, and this was defined as the lower-burden period [8]. Each period was limited to 3 years to ensure that the two periods were similar.

### Statistical analysis

The data are presented as a number (percentage) for categorical variables and a mean [standard deviation] for continuous variables. The baseline characteristics of the study population were compared using the Student’s *t*-test for continuous variables and the Chi-squared test for categorical variables.

All participants were followed up from the transplantation date to the tuberculosis diagnosis date, date of death, or end of study date, December 31, 2020—whichever came first. The tuberculosis’ cumulative incidence was estimated using a Fine and Gray sub-distribution hazard model, considering death as a competing risk event [11]. The cumulative incidence curves were investigated using tuberculosis burden, transplantation type (SOT, allogeneic HSCT, autologous HSCT), SOT subtype, and age at transplantation. They were compared using Gray’s test. Multiple comparisons with Bonferroni adjustments were performed. Risk factors associated with tuberculosis were examined using the Fine and Gray sub-distribution hazard model after considering death as a competing event. We ran a univariable model for each risk factor and a multivariable model to evaluate the effect of risk factors after adjusting for the confounding factors. Age, sex, diabetes mellitus, hypertension, asthma, chronic obstructive pulmonary disease, liver cirrhosis, chronic kidney disease, and previous tuberculosis history were considered risk factors, as reported previously [12]. The relative risk of developing tuberculosis among transplant recipients compared with the tuberculosis risk of the general population in South Korea was calculated using the SIR. SIR was the ratio of the observed to the expected number of tuberculosis cases. The expected numbers of tuberculosis cases were computed using sex-specific and 5-year-age-specific incidence rates in the general population. Confidence intervals (95% CIs) were calculated assuming a Poisson distribution at the 95% level [13]. We used data from the Korean Tuberculosis Surveillance System, a mandatory reporting system for confirmed tuberculosis cases, to compare tuberculosis incidence between transplant recipients and the general population [8]. All statistical analyses were performed using SAS Enterprise Guide version 7.1 (SAS Institute, Cary, NC, USA). Two-sided *P-*values < 0.05 were considered significant.

### Ethics

This study was reviewed and approved by the Institutional Review Board of Severance Hospital, Yonsei University College of Medicine, Seoul, Korea (Reg. No. 4-2020-0018). The requirement for informed consent was waived owing to the use of anonymous data.

## Results

### Characteristics of the study population

**Fig. 1:**
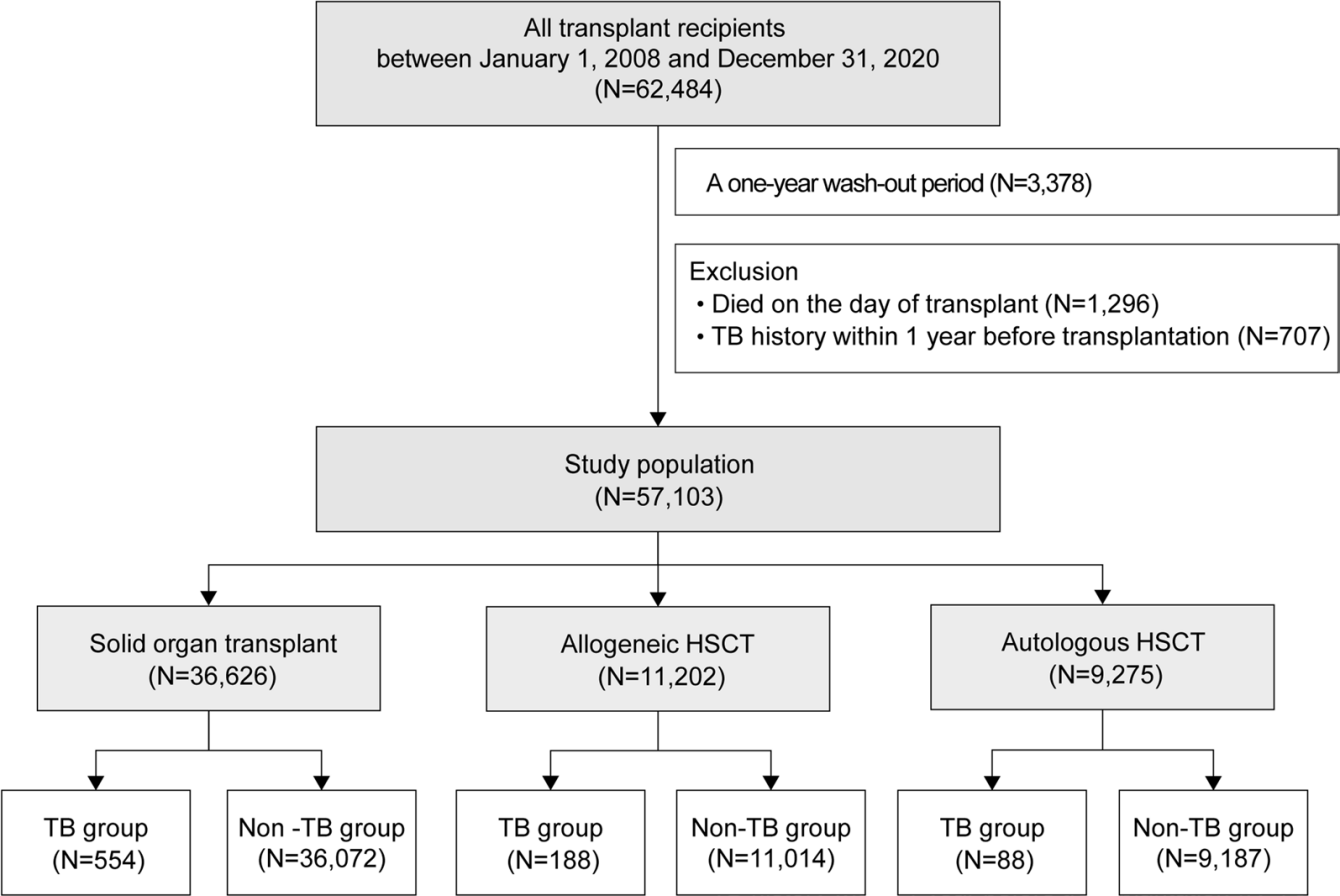
Flow chart of the study population. HSCT, hematopoietic stem cell transplantation; TB, tuberculosis

From 2008 to 2020, 62,484 insured individuals who had undergone transplantation were identified. Of these, 3,378 were excluded, and 57,103 were finally included in the analyses **(**Fig. 1). The male-to-female ratio was 1.6:1, and the mean follow-up duration was 5.30 [3.54] years. By age group, the 40–59-year-old group accounted for most of the participants at 55.7%, followed by the ≥ 60-year-old (18.5%), 20–39-year-old (17.3%), and < 20-year-old (8.5%) groups. According to transplant type, SOT accounted for 64.1%, allogeneic HSCT for 19.6%, and autologous HSCT for 16.2%. Among the recipients of SOTs, 57.1% had kidney transplantation (KT), 37.0% had liver transplantation (LT), 3.7% had heart transplantation (HT), 1.7% had lung transplantation, and 0.4% had others. Of these recipients, 3.0% had a tuberculosis history before transplantation. The other baseline characteristics of all study populations are presented in Table 1. The detailed baseline characteristics of SOT and HSCT recipients are presented in Additional file 6: Table S6 and Additional file 7: Table S7, respectively.

**Table 1:** Baseline characteristics of the study population

Characteristics	All transplantations			
	Total (n = 57,103)	TB group (n = 830)	Non-TB group (n = 56,273)	*P*-value
Age at transplant (years)				
0–19	4,851 (8.5)	30 (3.6)	4,821 (8.6)	< 0.001
20–39	9,884 (17.3)	128 (15.4)	9,756 (17.3)	
40–59	31,783 (55.7)	474 (57.1)	31,309 (55.6)	
> 60	10,585 (18.5)	198 (23.9)	10,387 (18.5)	
Sex				
Male	35,123 (61.5)	575 (69.3)	34,548 (61.4)	< 0.001
Female	21,980 (38.5)	255 (30.7)	21,725 (38.6)	
Duration of follow-up (years)	5.30 [3.54]	1.75 [2.03]	5.35 [3.53]	< 0.001
Comorbidities				
Diabetes mellitus	23,028 (40.3)	377 (45.4)	22,651 (40.3)	0.003
Hypertension	32,726 (57.3)	485 (58.4)	32,241 (57.3)	0.51
Asthma	7,841 (13.7)	107 (12.9)	7,734 (13.7)	0.48
COPD	3,186 (5.6)	42 (5.1)	3,144 (5.6)	0.51
Liver Cirrhosis	12,019 (21.0)	198 (23.9)	11,821 (21.0)	0.05
Chronic kidney disease	21,930 (38.4)	319 (38.4)	21,611 (38.4)	0.99
Solid cancer	12,696 (22.4)	200 (24.1)	12,496 (22.2)	0.19
Hematologic malignancy	12,547 (22.0)	174 (21.0)	12,373 (22.0)	0.48
Autoimmune disease	309 (0.5)	1 (0.1)	308 (0.5)	0.14
Charlson Comorbidity Index	5.48 [2.85]	5.89 [2.95]	5.48 [2.85]	< 0.001
Risk factors				
Previous TB History	1,741 (3.0)	37 (4.5)	1,704 (3.0)	0.02
Pulmonary TB	965 (1.7)	27 (3.2)	938 (1.7)	< 0.001
Extrapulmonary TB	1,024 (1.8)	15 (1.8)	1,009 (1.8)	0.98
Transplant type				
SOT	36,626 (64.1)	554 (66.7)	36,072 (64.1)	0.003
Allogeneic HSCT	11,202 (19.6)	188 (22.7)	11,014 (19.6)	0.03
Autologous HSCT	9,275 (16.2)	88 (10.6)	9,187 (16.3)	< 0.001
SOT transplant site				
Kidney^a^	20,904 (36.6)	296 (35.7)	20,608 (36.6)	0.57
Heart	1,373 (2.4)	19 (2.3)	1,354 (2.4)	0.83
Liver	13,568 (23.8)	224 (27.0)	13,344 (23.7)	0.03
Lung	600 (1.1)	11 (1.3)	589 (1.0)	0.44
Others^b^	181 (0.3)	4 (0.5)	177 (0.3)	0.39
Transplant year				
2009	3,422 (6.0)	99 (11.9)	3,323 (5.9)	< 0.001
2010	3,687 (6.5)	92 (11.1)	3,595 (6.4)	
2011	4,220 (7.4)	100 (12.0)	4,120 (7.3)	
2012	4,481 (7.8)	96 (11.6)	4,385 (7.8)	
2013	4,415 (7.7)	80 (9.6)	4,335 (7.7)	
2014	4,713 (8.3)	76 (9.2)	4,637 (8.2)	
2015	4,941 (8.7)	59 (7.1)	4,882 (8.7)	

Data are expressed as a number (percent) or a mean [standard deviation]

^a^Kidney includes kidney and kidney-pancreas transplantations

^b^Others include small bowel transplantation and pancreatic transplantation alone

COPD, chronic obstructive pulmonary disease; HSCT, hematopoietic stem cell transplantation; SOT, solid organ transplantation; TB, tuberculosis

### Incidence of active tuberculosis

**Fig. 2:**
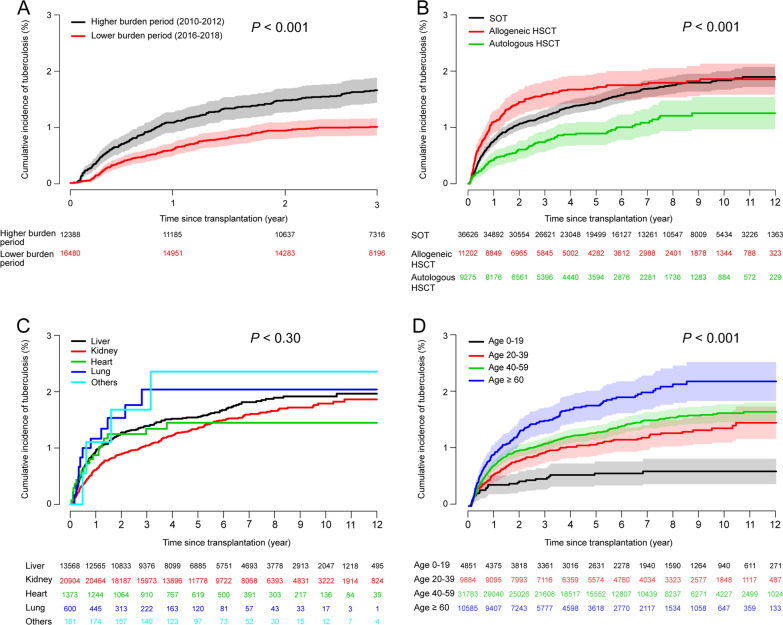
Kaplan–Meier curves for the cumulative incidence of tuberculosis. **a** Higher-burden period (2010–2012) and lower-burden period (2016–2018); **b** Transplant type; **c** SOT type; **d** Age. HSCT, hematopoietic stem cell transplantation; SOT, solid organ transplantation

During the follow-up period (mean [SD], 1.8 [2.0] years), tuberculosis occurred in 830 (1.5%) patients after transplantation. The overall cumulative incidence rate was 0.8% (95% CI 0.7–0.8) 1 year after transplantation, 1.1% (95% CI 1.0–1.1) at 2 years, 1.4% (95% CI 1.3–1.5) at 5 years, and 1.7% (95% CI 1.6–1.9) at 10 years. The cumulative incidence in the higher-burden period (2010–2012) was 1.7% 3 years after transplantation, significantly lower than that in the lower-burden period (2016–2018) (1.0%, *P* < 0.001) **(**Fig. 2a**).** Comparing tuberculosis incidence according to transplantation type, allogeneic HSCT had the highest tuberculosis incidence, followed by SOT. In contrast, autologous HSCT had the lowest rate (*P* < 0.001). Notably, the probability of developing tuberculosis among allogeneic HSCT recipients increased steeply up to 2 years after transplantation. Allogeneic HSCT and SOT differed in incidence up to 4 years after transplantation (1.7% for allogeneic HSCT and 1.4% for SOT, *P* = 0.026). However, there was almost no statistical difference thereafter, with 1.9% and 1.8% at 10 years for HSCT and SOT, respectively (*P* = 0.88). Tuberculosis incidence 2 years after transplantation was 1.5% in allogeneic HSCT recipients, 2.4 times higher than 0.6% in autologous HSCT recipients (*P* = 0.001) (Fig. 2b). By SOT, the highest cumulative incidence rate 5 years after transplantation occurred among those who had other transplantation types (2.4%), including small intestine and pancreas transplantations alone, followed by lung transplantation (2.0%), LT (1.6%), HT (1.4%), and KT (1.3%); however, the difference was not significant (Fig. 2c). Based on age group, the incidence of tuberculosis increased with age (*P* < 0.001). The 10-year cumulative incidence rate for those aged ≥ 60 years was 2.4%, which was 3.6 times higher than that for those aged 0–9 years (0.7%, *P* < 0.001) (Fig. 2d). In the SOT and allogeneic HSCT groups, tuberculosis incidence increased significantly with age, similar to the trend in the overall transplantation group. However, no difference in tuberculosis incidence by age group was observed in the autologous HSCT group. Based on sex, male recipients had a significantly higher tuberculosis risk than females, regardless of transplantation type (*P* < 0.001).

Among all active tuberculosis cases, pulmonary tuberculosis accounted for 64.7% (n = 537) and extrapulmonary tuberculosis accounted for 34.5% (n = 286). There was no significant difference in the incidence of pulmonary and extrapulmonary tuberculosis according to transplantation type (Additional file 8: Table S8).

### Risk factors for tuberculosis and mortality

Table 2 summarizes the tuberculosis risk factors for transplant recipients. In the multivariate analysis, male sex (hazard ratio [HR]: 1.4, 95% CI 1.2–1.6), older age ≥ 60 years (HR 1.7, 95% CI 1.3–2.1; reference, 20–39-year-old group), tuberculosis history (HR 1.5, 95% CI 1.1–2.1), and LT and allogeneic HSCT (HR 1.7, 95% CI 1.04–2.8; HR 2.1, 95% CI 1.4–3.3, respectively; reference transplantation, KT) were associated with increased risk of active tuberculosis. Similar risk factors for tuberculosis were identified in the SOT (Additional file 9: Table S9) and HSCT (Additional file 10: Table S10) subgroup analyses.

**Table 2:** Risk factors for active tuberculosis development after transplantation

Risk factors		Univariate		Multivariate	
		HR (95% CI)	*P*-value	HR (95% CI)	*P*-value
Age (years)	0–19	0.48 (0.32, 0.71)	< 0.001	0.46 (0.31, 0.69)	< 0.001
	20–39	1 (ref.)		1 (ref.)	
	40–59	1.18 (0.97, 1.44)	0.09	1.21 (0.99, 1.48)	0.06
	> 60	1.58 (1.26, 1.97)	< 0.001	1.65 (1.30, 2.08)	< 0.001
Sex	M	1.412 (1.22, 1.64)	< 0.001	1.38 (1.19,1.61)	< 0.001
	F	1 (ref.)		1 (ref.)	
Diabetes mellitus	Yes	1.26 (1.10, 1.44)	0.001	1.120 (0.97, 1.29)	0.13
	No	1 (ref.)		1 (ref.)	
Hypertension	Yes	1.07 (0.93, 1.22)	0.37	0.97 (0.82, 1.15)	0.73
	No	1 (ref.)		1 (ref.)	
Asthma	Yes	0.93 (0.76, 1.14)	0.49	0.97 (0.78, 1.20)	0.77
	No	1 (ref.)		1 (ref.)	
COPD	Yes	0.99 (0.73, 1.35)	0.95	0.85 (0.62, 1.18)	0.34
	No	1 (ref.)		1 (ref.)	
Liver Cirrhosis	Yes	1.17 (1.00, 1.37)	0.05	0.90 (0.67, 1.20)	0.46
	No	1 (ref.)		1 (ref.)	
Chronic kidney disease	Yes	1.00 (0.87, 1.15)	0.96	1.46 (0.98, 2.19)	0.07
	No	1 (ref.)		1 (ref.)	
Previous TB history	Yes	1.54 (1.11, 2.14)	0.010	1.48 (1.06, 2.07)	0.02
	No	1 (ref.)		1 (ref.)	
Transplantation	Liver	1.17 (0.98, 1.39)	0.08	1.71 (1.04, 2.80)	0.034
	Kidney	1 (ref.)		1 (ref.)	
	Heart	1.00 (0.63, 1.59)	0.99	1.39 (0.80, 2.41)	0.25
	Lung	1.42 (1.78, 2.60)	0.25	1.88 (1.90, 3.92)	0.09
	Others	1.59 (0.59, 4.25)	0.36	2.55 (0.89, 7.30)	0.08
	Allogeneic HSCT	1.20 (1.00, 1.44)	0.05	2.14 (1.37, 3.34)	< 0.001
	Autologous HSCT	0.68 (0.54, 0.87)	0.002	1.01 (0.64, 1.57)	0.97

CI, confidence interval; COPD, chronic obstructive pulmonary disease; HR, hazard ratio; HSCT, hematopoietic stem cell transplantation; SOT, solid organ transplantation; TB, tuberculosis

Among transplant recipients with tuberculosis, the all-cause mortality rate during the study period was 27.0%, significantly higher than that of transplant recipients without tuberculosis (17.1%, *P* < 0.001).

### Comparison with the general population

**Fig. 3:**
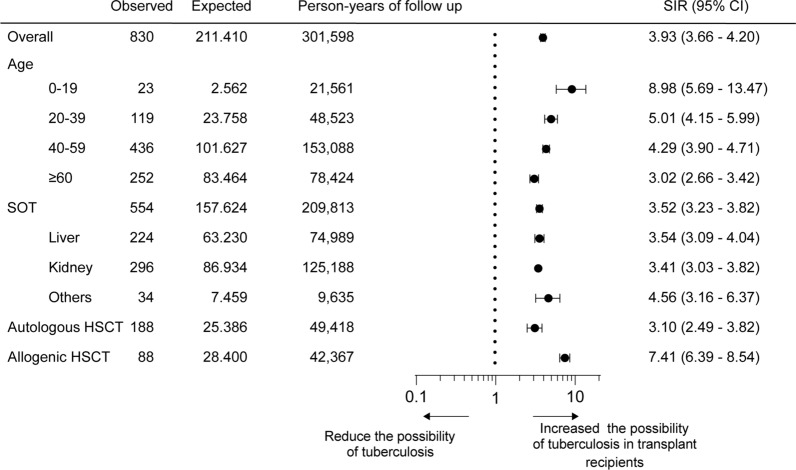
Age-adjusted standardized incidence ratios. SIR, standardized incidence rate; CI, confidence interval; HSCT, hematopoietic stem cell transplantation; SOT, solid organ transplantation

The overall age-adjusted SIR result revealed that transplant recipients had 3.9 times higher tuberculosis risk than the general Korean population (SIR: 3.9, 95% CI 3.7–4.2). The SIR of transplanted recipients aged < 20 years was 9.0 (95% CI 5.7–13.5), the highest among all age groups. Meanwhile, the SIR of the 20–39-year-old group was 5.0 (95% CI 4.2–6.00), that of the 40–59-year-old group was 4.3 (95% CI 3.9–4.7), and that of the 60-year-old group was 3.0 (95% CI 2.7–3.4). Based on the transplantation type, the SIR of LT recipients was 3.5 times (95% CI 3.1–4.0); KT, 3.4 times (95% CI 3.0–3.8); autologous HSCT, 7.4 times (95% CI 6.4–8.5); and allogeneic HSCT, 3.1 times (95% CI 2.5–3.8) more than that of the general population (Fig. 3).

## Discussion

We revealed the effect of exogenous exposure on tuberculosis occurrence in high-risk transplant recipients by comparing different incidence rates in Korea. In addition, the risk of tuberculosis varied by up to 2.4 times depending on the transplant type (SOT or HSCT). Additionally, the risk differed depending on the type of SOT. Also, tuberculosis risk differed by the recipient’s age at the time of transplantation; the lowest risk was observed in the pediatric population, but their SIR was the highest.

The 10-year cumulative tuberculosis incidence among transplant recipients in this study (1.7%) was comparable to the cumulative incidence in previous studies in countries/regions with a similar tuberculosis prevalence (50–100 cases per 100,000) [14, 15]. In Taiwan (55–67 cases per 100,000 of the general population during 2006–2011), the 10-year cumulative incidence of tuberculosis was 3.5% among 2,040 adult HSCT recipients [15]. In addition, the 5-year cumulative tuberculosis incidence in adult SOT recipients in 2011–2016 in Korea was 1.9% [16]. Our results are significant because these large-scale population-based data provide a more specific range of active tuberculosis incidence among transplant recipients in countries with a moderate prevalence of endemic tuberculosis [17]. Moreover, this study’s case definitions and criteria can be applied in future population-based studies in other countries.

The most notable finding in the present study is that active tuberculosis incidence in transplant recipients decreased by almost half (58.8%; 1.7% vs. 1.0% 3 years after transplantation), as the tuberculosis incidence rate in the general Korean population decreased from approximately 100 to 70 per 100,000. In the Spanish Network of Infection in Transplantation cohort study conducted among 4,388 SOT recipients in Spain, where tuberculosis incidence was low, 95% of patients with tuberculosis were infected within 1 year after transplantation [6]. Comparatively, our data revealed a more linear pattern for active tuberculosis after transplantation. This suggests that tuberculosis exposure after transplantation is as essential as the reactivation of latent tuberculosis in high-risk populations in endemic countries/regions. Transplant recipients are at risk of tuberculosis at both early and later transplantation periods. Therefore, latent tuberculosis screening in the pre-transplantation period, as recommended in the current guidelines for transplantation-associated infection, and early suspicion and aggressive diagnostic testing for tuberculosis in the late post-transplantation period in moderate-to-highly endemic countries/regions are necessary.

Our study also directly compared allogeneic HSCT, autologous HSCT, and SOT. Allogeneic recipients of HSCT had the highest cumulative incidence of active tuberculosis, followed by recipients of SOT and autologous HSCT. In a previous study, tuberculosis incidence was 10 times higher in recipients of SOT than in those of HSCT [7, 18, 19], possibly due to long-term immunosuppressant use. However, allogeneic and autologous HSCT should be considered separately [19]. The profound and prolonged deficiency of cell-mediated immunity in allogeneic HSCT due to pre-transplant conditioning therapy, immunosuppression after transplantation, and graft-versus-host disease is a major factor in tuberculosis development after transplantation. In addition, the fact that tuberculosis generally occurs within 1 year after transplantation is a more critical factor in the degree of immunosuppression than the long-term use of immunosuppressants that have been previously proposed. Previous studies have reported that tuberculosis risk differs according to the type of transplanted organ and is particularly high in recipients of lung transplants [6, 16, 18, 20]. Our cohort also displayed similar tendencies, with more cases of tuberculosis among those who underwent lung transplantation, followed by those who underwent LT, KT, and HT. Individuals with small intestine and pancreatic transplantations—classified as other transplantations—also had a high incidence, probably because of the relatively few transplants.

Interestingly, children had the lowest tuberculosis incidence among transplant recipients, but they had the highest tuberculosis risk compared to the general population of children of the same age. Previous literature on tuberculosis in pediatric recipients of SOT and HSCT is sparse, and the underlying reason is not apparent [21]. One possible explanation could be that pediatric transplant recipients have a relatively low tuberculosis exposure risk compared to adult or elderly transplant recipients (shorter exposure during lifetime and lower tuberculosis incidence in Korea in recent years compared to the past), which could result in lower age-specific transplant tuberculosis rates. However, a higher incidence of active tuberculosis than children of the same age should be noted. Therefore, upon exposure to tuberculosis, aggressive screening and treatment for latent tuberculosis in immunocompromised children are warranted [22].

This study has few limitations. First, we could not evaluate the effects of immunosuppressive agents. Second, information on latent tuberculosis was unavailable in our dataset. National guidelines for the diagnosis and treatment of latent tuberculosis have been suggested since 2014, but there is a lack of assessment regarding its impact on transplant patients. The development and treatment of latent tuberculosis after transplantation may have affected active tuberculosis development. Third, although mortality was higher in the tuberculosis group than in the non-tuberculosis group, the Charlson comorbidity index was also higher; therefore, it was challenging to directly explain the high mortality among those with tuberculosis.

Nevertheless, our study has several strengths. First, to the best of our knowledge, this is the largest population-based study evaluating active tuberculosis incidence among transplant recipients compared with that among the age- and sex-adjusted general population. Second, by comparing various transplants according to the same definition and criteria, we could measure the relative risk according to transplant type and organ transplantation. Finally, this study is relevant because it measured tuberculosis development risk in pediatric transplant recipients previously neglected in research.

## Conclusions

Tuberculosis development risk after transplantation is several times higher than that in the general population and is influenced by community prevalence. Furthermore, the prevalence by country, transplant type, and age should be considered to establish an appropriate tuberculosis prevention strategy for these high-risk groups.

### Supplementary Information

**Additional file 1: Table S1.** The annual reported number and incidence of tuberculosis in Korea, 2008-2020.

**Additional file 2: Table S2.** The case number of transplantations by year in Korea, 2008-2020.

**Additional file 3: Table S3.** HIRA code for Transplantation.

**Additional file 4: Table S4.** Drug ATC codes used to define tuberculosis.

**Additional file 5: Table S5.** ICD-10 Code for tuberculosis and co-variables.

**Additional file 6: Table S6.** Baseline characteristics of patients with SOT.

**Additional file 7: Table S7.** Baseline characteristics of patients with HSCT.

**Additional file 8: Table S8.** Pulmonary and extrapulmonary tuberculosis according to transplant type.

**Additional file 9: Table S9.** Risk factors associated with the development of TB after SOT.

**Additional file 10: Table S10.** Risk factors associated with the development of TB after HCST.

## Data Availability

Data are available through the Health Insurance Review & Assessment Service (https://opendata.hira.or.kr).

## Abbreviations

CI: Confidence interval
HIRA: Health insurance review and assessment
HR: Hazard ratio
HSCT: Hematopoietic stem cell transplant
HT: Heart transplantation
KT: Kidney transplantation
LT: Liver transplantation
NHIS: National Health Insurance Service
SOT: Solid organ transplant
SIR: Standardized incidence ratio
