# Supplementary material for: Changes in tuberculosis risk after transplantation in the setting of decreased community tuberculosis incidence: a national population-based study, 2008–2020
Source: Ann Clin Microbiol Antimicrob. 2024 Jan 3;23:1. doi: 10.1186/s12941-023-00661-4 (PMC10765802; doi:10.1186/s12941-023-00661-4)
Supplement: Supplementary file 1 — Additional file 1: Table S1. The annual reported number and incidence of tuberculosis in Korea, 2008-2020. [file 12941_2023_661_MOESM1_ESM.docx]

**Supplementary Table 1. The annual reported number and incidence of tuberculosis in Korea, 2008-2020**

|  | **2008** | **2009** | **2010** | **2011** | **2012** | **2013** | **2014** | **2015** | **2016** | **2017** | **2018** | **2019** | **2020** |
| --- | --- | --- | --- | --- | --- | --- | --- | --- | --- | --- | --- | --- | --- |
| **Newly diagnosed TB** | 34,157 | 35,845 | 36,305 | 39,557 | 39,545 | 36,089 | 34,869 | 32,181 | 30,892 | 28,161 | 26,433 | 23,821 | 19,933 |
| **Pulmonary TB** |  | 28,922 | 28,176 | 30,100 | 31,075 | 28,720 | 27,906 | 25,550 | 24,696 | 22,314 | 20,883 | 18,765 | 15,221 |
| **Extrapulmonary TB** |  | 6,923 | 8,129 | 9,457 | 8,470 | 7,369 | 6,963 | 6,631 | 6,196 | 5,847 | 5,550 | 5,056 | 4,712 |
| **Relapse TB** | 7,917 | 8,655 | 8,794 | 9,419 | 7,750 | 8,128 | 6,254 | 6,211 | 6,087 | 5,637 | 5,243 | 4,679 | 3,709 |
| **Unclassified TB** | 2,100 | 2,802 | 3,002 | 1,515 | 2,237 | 1,075 | 1,713 | 2,029 | 1,704 | 1,636 | 1,465 | 1,352 | 1,320 |
| **Total TB** | 44,174 | 47,302 | 48,101 | 50,491 | 49,532 | 45,292 | 43,088 | 40,847 | 39,245 | 36,044 | 33,796 | 30,304 | 25,350 |
| **Incidence of**  **newly diagnosed TB^a^** | 69.1 | 72.2 | 72.8 | 78.9 | 78.5 | 71.4 | 68.7 | 63.2 | 60.4 | 55 | 51.5 | 46.4 | 38.8 |
| **Incidence of**  **total TB^a^** | 89.4 | 95.3 | 96.4 | 100.8 | 98.4 | 89.6 | 84.9 | 80.2 | 76.8 | 70.4 | 65.9 | 59.0 | 49.4 |

**^a^**incidence per 100,000 people.

Abbreviations: TB, tuberculosis
