# Supplementary material for: Changes in tuberculosis risk after transplantation in the setting of decreased community tuberculosis incidence: a national population-based study, 2008–2020
Source: Ann Clin Microbiol Antimicrob. 2024 Jan 3;23:1. doi: 10.1186/s12941-023-00661-4 (PMC10765802; doi:10.1186/s12941-023-00661-4)
Supplement: Supplementary file 2 — Additional file 2: Table S2. The case number of transplantations by year in Korea, 2008-2020. [file 12941_2023_661_MOESM2_ESM.docx]

**Supplementary Table 2. The case number of transplantations by year in Korea, 2008-2020**

|  | **2008** | **2009** | **2010** | **2011** | **2012** | **2013** | **2014** | **2015** | **2016** | **2017** | **2018** | **2019** | **2020** | **Total** |
| --- | --- | --- | --- | --- | --- | --- | --- | --- | --- | --- | --- | --- | --- | --- |
| **SOT** |  |  |  |  |  |  |  |  |  |  |  |  |  |  |
| **Liver** | 896 | 936 | 1,005 | 1,109 | 1,188 | 1,078 | 1,167 | 1,297 | 1,357 | 1,375 | 1,339 | 1,447 | 1,082 | 15,276 |
| **Kidney**^*^ | 1,145 | 1,214 | 1,259 | 1,594 | 1,754 | 1,704 | 1,767 | 1,855 | 2,172 | 2,096 | 2,061 | 2,236 | 1,617 | 22,474 |
| **Heart** | 82 | 62 | 69 | 85 | 100 | 113 | 107 | 136 | 148 | 172 | 165 | 176 | 122 | 1,537 |
| **Lung** | 5 | 8 | 15 | 28 | 30 | 32 | 44 | 51 | 77 | 83 | 79 | 125 | 98 | 675 |
| **Others^†^** | 2 | 5 | 3 | 6 | 6 | 20 | 23 | 23 | 32 | 22 | 16 | 20 | 8 | 186 |
| **Total** | 2,130 | 2,225 | 2,351 | 2,822 | 3,078 | 2,947 | 3,108 | 3,362 | 3,786 | 3,748 | 3,660 | 4,004 | 2,927 | 40,148 |
| **HSCT** |  |  |  |  |  |  |  |  |  |  |  |  |  |  |
| **Allogeneic** | 696 | 768 | 839 | 933 | 897 | 893 | 978 | 963 | 1,013 | 997 | 1,078 | 1,224 | 1,037 | 12,316 |
| **Autologous** | 552 | 595 | 639 | 622 | 693 | 731 | 809 | 799 | 864 | 919 | 930 | 986 | 881 | 10,020 |
| **Total** | 1,248 | 1,363 | 1,478 | 1,555 | 1,590 | 1,624 | 1,787 | 1,762 | 1,877 | 1,916 | 2,008 | 2,210 | 1,918 | 22,336 |
| **All transplantation** | 3,378 | 3,588 | 3,829 | 4,377 | 4,668 | 4,571 | 4,895 | 5,124 | 5,663 | 5,664 | 5,668 | 6,214 | 4,845 | 62,484 |

*Kidney includes kidney and kidney-pancreas transplantation. **^†^**Others include small bowel transplantation and pancreas transplantation alone.

Abbreviations: SOT, solid organ transplantation; HSCT, hematopoietic stem cell transplantation
