# Supplementary material for: Changes in tuberculosis risk after transplantation in the setting of decreased community tuberculosis incidence: a national population-based study, 2008–2020
Source: Ann Clin Microbiol Antimicrob. 2024 Jan 3;23:1. doi: 10.1186/s12941-023-00661-4 (PMC10765802; doi:10.1186/s12941-023-00661-4)
Supplement: Supplementary file 3 — Additional file 3: Table S3. HIRA code for Transplantation. [file 12941_2023_661_MOESM3_ESM.docx]

**Supplementary Table 3. HIRA code for Transplantation**

| **Transplantation** | **Code** | **Description** |
| --- | --- | --- |
| **Liver** | Q8040 | Liver Transplantation from Cadaver Donor, Total |
|  | Q8041 | Liver Transplantation from Cadaver Donor, Total, Right Trisection |
|  | Q8042 | Liver Transplantation from Cadaver Donor, Total, Simple Right Lobe |
|  | Q8043 | Liver Transplantation from Cadaver Donor, Split, Left Lateral Segment |
|  | Q8044 | Liver Transplantation from Cadaver Donor, Split, Left Lobe |
|  | Q8045 | Partial Liver Transplantation from Living Donor, Left Lateral Segment |
|  | Q8046 | Partial Liver Transplantation from Living Donor, Left Lobe |
|  | Q8047 | Partial Liver Transplantation from Living Donor, Right Lobe |
|  | Q8048 | Partial Liver Transplantation from Living Donor, Extended Right Lobe |
|  | Q8049 | Partial Liver Transplantation from Living Donor, Modified Right Lobe |
|  | Q8050 | Partial Liver Transplantation from Living Donor, Dural Graft |
| **Kidney** | R3280 | Renal Transplantation |
| **Heart** | Q8080 | Heart Transplantation |
| **Lung** | Q8101 | Lung Transplantation, Single |
|  | Q8102 | Lung Transplantation, Double |
| **Small Bowel** | Q8121 | Small Bowel Transplantation from Cadaver Donor Total |
|  | Q8122 | Small Bowel Transplantation from Cadaver Donor Partial |
|  | Q8123 | Partial Small Bowel Transplantation from Living Donor |
| **Pancreas** | Q8061 | Pancreas Transplantation Partial |
|  | Q8062 | Pancreas Transplantation Pancreas and Duodenum |
| **Allogeneic HSCT** | X5131 | Hemopoietic Cell Transplantation, Bone marrow, Allogenic |
|  | X5133 | Hemopoietic Cell Transplantation, Blood-Derived hematopoietic progenitor cell, Allogenic |
|  | X5135 | Hemopoietic Cell Transplantation, Cord Blood, Allogenic |
| **Autologous HSCT** | X5132 | Hemopoietic Cell Transplantation, Bone marrow, Autologous |
|  | X5134 | Hemopoietic Cell Transplantation, Blood-Derived hematopoietic progenitor cell, Autologous |
|  | X5136 | Hemopoietic Cell Transplantation, Cord Blood, Autologous |

Abbreviations: HIRA, Health Insurance Review & Assessment; HSCT, hematopoietic stem cell transplantation
