# Supplementary material for: Changes in tuberculosis risk after transplantation in the setting of decreased community tuberculosis incidence: a national population-based study, 2008–2020
Source: Ann Clin Microbiol Antimicrob. 2024 Jan 3;23:1. doi: 10.1186/s12941-023-00661-4 (PMC10765802; doi:10.1186/s12941-023-00661-4)
Supplement: Supplementary file 4 — Additional file 4: Table S4. Drug ATC codes used to define tuberculosis. [file 12941_2023_661_MOESM4_ESM.docx]

**Supplementary Table 4. Drug ATC codes used to define tuberculosis**

|  | **Drug ATC code** | **Description** |
| --- | --- | --- |
| **Age under 20** | 155602ATB | ethambutol |
|  | 155605ATB | ethambutol |
|  | 223901ACH | rifampicin |
|  | 223902ACH | rifampicin |
|  | 223902ATB | rifampicin |
|  | 223903ATB | rifampicin |
|  | 223904ATB | rifampicin |
|  | 364401ACH | rifabutin |
|  | 380200ATB | rifampicin and isoniazid |
|  | 519500ATB | rifampicin, pyrazinamide, ethambutol and isoniazid |
| **Age above 20** | 155602ATB | ethambutol |
|  | 155605ATB | ethambutol |
|  | 221201ATB | pyrazinamide |
|  | 221202ATB | pyrazinamide |
|  | 519500ATB | rifampicin, pyrazinamide, ethambutol and isoniazid |
