# Supplementary material for: Changes in tuberculosis risk after transplantation in the setting of decreased community tuberculosis incidence: a national population-based study, 2008–2020
Source: Ann Clin Microbiol Antimicrob. 2024 Jan 3;23:1. doi: 10.1186/s12941-023-00661-4 (PMC10765802; doi:10.1186/s12941-023-00661-4)
Supplement: Supplementary file 5 — Additional file 5: Table S5. ICD-10 Code for tuberculosis and co-variables. [file 12941_2023_661_MOESM5_ESM.docx]

**Supplementary Table 5. ICD-10 Code for tuberculosis and co-variables**

| **Disease** | **ICD-10 code** |
| --- | --- |
| **Tuberculosis** |  |
| Pulmonary TB | A15.0, A15.1, A15.2, A15.3, A15.5, A16.0, A16.1, A16.2, A16.4, A19 |
| Extrapulmonary TB | A15.4, A15.6, A15.8, A15.9, A16.3, A16.5, A16.7, A16.8, A16.9, A17, A18 |
| MDR-TB | U84.30 |
| XDR-TB | U84.31 |
| Latent TB | Z22.7 |
| **Underlying Disease entities for HSCT** | |
| AML | C92.0, C92.4, C92.6, C92.8 |
| ALL | C91.0 |
| CML | C92.1, C92.2 |
| Lymphoma | C81, C82, C83, C84, C85, C86, |
| Multiple myeloma | C90 |
| Severe aplastic anemia | D60-D64 |
| MDS | D46 |
| Others | C88, C94, C95, C96, C91.1-91.9, C92.3, C92.7, C92.9 |
| **Comorbidities** |  |
| Diabetes Mellitus | E10, E11, E12, E13, E14 |
| Hypertension | I10, I11, I12, I13, I14, I15 |
| Asthma | J45 |
| COPD | J44 |
| Liver Cirrhosis | K74, K70.3, P78.8, K76.1, K71.7 |
| Chronic kidney disease | N18 |
| Post gastrectomy | K91.1, Z90.3 |
| HIV | B20.x-B22.x, B24.x |
| Solid cancer | C00-C80, C97 |
| Hematologic malignancy | C81-C96 |
| Autoimmune disease | G61.0, M05, M06, M08.0, M08.2-M08.9, M30-M36, D510, D59.0, D59.1, D86, E063, E05.0, E10, E27.1, E27.2, G35, G70.0, I02, I05-I09, K50, K51, K74.3, K75.4, K90.0, L10.0, L40.5, M070-M073, M09.0, L93.0, L94.0-L94.3, L95, M02.3, M30.0, M31.3, M31.5, M35.3, M35.2, M45.9, M08.1 |
| **Charlson comorbidity index** |  |
| Myocardial infarction | I21.x, I22.x, I25.2 |
| Congestive heart failure | I09.9,I11.0, I13.0, I13.2, I25.5, I42.0, I42.5-I42.9, I43.x, I50.x, P29.0 |
| Peripheral vascular disease | I70.x, I71.x, I73.1, I73.8, I73.9, I77.1, I79.0, I79.2, K55.1, K55.8, K55.9, Z95.8, Z95.9 |
| Cerebrovascular disease | G45.x, G46.x, H34.0, I60.x-I69.x |
| Dementia | F00.x-F03.x, F05.1, G30.x, G31.1 |
| Chronic pulmonary disease | I27.8, I27.9, J40.x-J47.x, J60.x-J67.x, J68.4, J70.1, J70.3 |
| Rheumatic disease | M05.x, M06.x, M31.5, M32.x-M34.x, M35.1, M35.3, M36.0 |
| Peptic ulcer disease | K25.x-K28.x |
| Mild liver disease | B18.x, K70.0-K70.3, K70.9, K71.3-K71.5, K71.7, K73.x, K74.x, K76.0, K76.2-K76.4, K76.8, K76.9, Z94.4 |
| Diabetes without chronic complication | E10.0, E10.l, E10.6, E10.8, E10.9, E11.0, E11.1, E11.6, E11.8, E11.9, E12.0, E12.1, E12.6, E12.8, E12.9, E13.0, E13.1, E13.6, E13.8, E13.9, E14.0, E14.1, E14.6, E14.8, E14.9 |
| Diabetes with chronic complication | E10.2-E10.5, E10.7, E11.2-E11.5, E11.7, E12.2-E12.5, E12.7, E13.2-E13.5, E13.7, E14.2-E14.5, E14.7 |
| Hemiplegia | G04.1, G11.4, G80.1, G80.2, G81.x, G82.x, G83.0-G83.4, G83.9 |
| Renal disease | I12.0, I13.1, N03.2-N03.7, N05.2-N05.7, N18.x, N19.x, N25.0, Z49.0-Z49.2, Z94.0, Z99.2 |
| Any malignancy, including lymphoma and leukemia, except malignant neoplasm of skin | C00.x-C26.x, C30.x-C34.x, C37.x-C41.x, C43.x, C45.x-C58.x, C60.x-C76.x, C81.x-C85.x, C88.x, C90.x-C97.x |
| Moderate or severe liver disease | I85.0, I85.9, I86.4, I98.2, K70.4, K71.1, K72.1, K72.9, K76.5, K76.6, K76.7 |
| Metastatic solid tumor | C77.x-C80.x |
| AIDS/HIV | B20.x-B22.x, B24.x |

Abbreviations: AIDS, acquired immune deficiency syndrome; ALL, acute lymphoblastic leukemia; AML, acute myeloid leukemia; CML, chronic myelogenous leukemia; COPD, chronic obstructive pulmonary disease; HIV, Human immunodeficiency virus; MDS, myelodysplastic syndrome; Multidrug-resistant tuberculosis, MDR-TB; TB, tuberculosis; XDR-TB, Extensively drug-resistant tuberculosis
