# Supplementary material for: Changes in tuberculosis risk after transplantation in the setting of decreased community tuberculosis incidence: a national population-based study, 2008–2020
Source: Ann Clin Microbiol Antimicrob. 2024 Jan 3;23:1. doi: 10.1186/s12941-023-00661-4 (PMC10765802; doi:10.1186/s12941-023-00661-4)
Supplement: Supplementary file 6 — Additional file 6: Table S6. Baseline characteristics of patients with SOT. [file 12941_2023_661_MOESM6_ESM.docx]

**Supplementary Table 6.** **Baseline characteristics of patients with SOT**

|  | **SOT** | | | |
| --- | --- | --- | --- | --- |
|  | **Total** | **TB group** | **Non-TB group** | ***p*-value** |
|  | (n=36,626) | (n=554) | (n=36.072) |  |
| **Age at transplant** |  |  |  |  |
| 0 to 19 years | 1,325 (3.6) | 2 (0.4) | 1,323 (3.7) | <0.001 |
| 20 to 39 years | 6,005 (16.4) | 69 (12.5) | 5,936 (16.5) |  |
| 40 to 59 years | 22,264 (60.8) | 334 (60.3) | 21,930 (60.8) |  |
| over 60 years | 7,032 (19.2) | 149 (26.9) | 6,883 (19.1) |  |
| **Sex** |  |  |  |  |
| Male | 23,440 (64.0) | 393 (70.9) | 23,047 (63.9) | <0.001 |
| Female | 13,186 (36.0) | 161 (29.1) | 13,025 (36.1) |  |
| **Duration of follow-up, year** | 5.74 ± 3.44 | 1.90 ± 2.13 | 5.80 ± 3.42 | <0.001 |
| **Comorbidities** |  |  |  |  |
| Diabetes mellitus | 17,524 (47.8) | 291 (52.5) | 17,233 (47.8) | 0.026 |
| Hypertension | 26,550 (72.5) | 393 (70.9) | 26,157 (72.5) | 0.41 |
| Asthma | 3,658 (10.0) | 60 (10.8) | 3,598 (10.0) | 0.50 |
| COPD | 1,291 (3.5) | 20 (3.6) | 1,271 (3.5) | 0.91 |
| Liver Cirrhosis | 11,664 (31.8) | 195 (35.2) | 11,469 (31.8) | 0.09 |
| Chronic kidney disease | 21,313 (58.2) | 306 (55.2) | 21,007 (58.2) | 0.16 |
| Solid cancer | 8,161 (22.3) | 140 (25.3) | 8,021 (22.2) | 0.09 |
| Hematologic malignancy | 105 (0.2) | 4 (0.7) | 101 (0.3) | 0.08 |
| Autoimmune disease | 87 (0.2) | 0 | 87 (0.2) | 0.65 |
| **Charlson comorbidity index** | 5.68 ± 2.69 | 6.26 ± 2.78 | 5.67 ± 2.69 | <0.001 |
| **Risk factors** |  |  |  |  |
| Previous TB History | 1,319 (3.6) | 26 (4.7) | 1,293 (3.6) | 0.17 |
| Pulmonary TB | 735 (2.0) | 17 (3.1) | 718 (2.0) | 0.07 |
| Extrapulmonary TB | 774 (2.1) | 13 (2.3) | 761 (2.1) | 0.70 |
| **Transplant site of SOT** |  |  |  | <0.001 |
| Kidney* | 20,904 (57.1) | 296 (53.4) | 20,608 (57.1) | 0.08 |
| Heart | 1,373 (3.7) | 19 (3.4) | 1,354 (3.8) | 0.69 |
| Liver | 13,568 (37.0) | 224 (40.4) | 13,344 (37.0) | 0.10 |
| Lung | 600 (1.6) | 11 (2.0) | 589 (1.6) | 0.52 |
| Others ^†^ | 181 (0.5) | 4 (0.7) | 177 (0.5) | 0.36 |
| **Transplant year** |  |  |  |  |
| 2009 | 2,106 (5.8) | 58 (10.5) | 2,408 (5.7) | <0.001 |
| 2010 | 2,248 (6.1) | 51 (9.2) | 2,197 (6.1) |  |
| 2011 | 2,720 (7.4) | 73 (13.2) | 2,647 (7.3) |  |
| 2012 | 2,956 (8.1) | 64 (11.6) | 2,892 (8.0) |  |
| 2013 | 2,847 (7.8) | 59 (10.7) | 2,788 (7.7) |  |
| 2014 | 2,994 (8.2) | 49 (8.8) | 2,945 (8.2) |  |
| 2015 | 3,227 (8.8) | 42 (7.6) | 3,185 (8.8) |  |
| 2016 | 3,651 (10.0) | 63 (11.4) | 3,588 (9.9) |  |
| 2017 | 3,618 (9.9) | 38 (6.9) | 3,580 (9.9) |  |
| 2018 | 3,544 (9.7) | 24 (4.3) | 3,520 (9.8) |  |
| 2019 | 3,880 (10.6) | 25 (4.5) | 3,855 (10.7) |  |
| 2020 | 2,835 (7.7) | 8 (1.4) | 2,827 (7.8) |  |
| **Outcome** |  |  |  |  |
| Death | 3,444 (9.4) | 90 (16.2) | 3,354 (9.3) | <0.001 |

Note: Data are expressed number (percent) or mean ± standard deviation.

*Kidney includes kidney and kidney-pancreas transplantation. ^†^Others include small bowel transplantation and pancreas transplantation alone.

Abbreviations: COPD, chronic obstructive pulmonary disease; SOT, solid organ transplantation; TB, tuberculosis
