# Supplementary material for: Changes in tuberculosis risk after transplantation in the setting of decreased community tuberculosis incidence: a national population-based study, 2008–2020
Source: Ann Clin Microbiol Antimicrob. 2024 Jan 3;23:1. doi: 10.1186/s12941-023-00661-4 (PMC10765802; doi:10.1186/s12941-023-00661-4)
Supplement: Supplementary file 7 — Additional file 7: Table S7. Baseline characteristics of patients with HSCT. [file 12941_2023_661_MOESM7_ESM.docx]

**Supplementary Table 7.** **Baseline characteristics of patients with HSCT**

|  | **HSCT** | | | |
| --- | --- | --- | --- | --- |
|  | **Total** | **TB group** | **Non-TB group** | ***p*-value** |
|  | (n=20,477) | (n=276) | (n=20,201) |  |
| **Age at transplant** |  |  |  |  |
| 0 to 19 years | 3,526 (17.2) | 28 (10.1) | 3,498 (17.3) | 0.018 |
| 20 to 39 years | 3,879 (18.9) | 59 (21.4) | 3,820 (18.9) |  |
| 40 to 59 years | 9,519 (46.5) | 140 (50.7) | 9,379 (46.4) |  |
| over 60 years | 3,553 (17.4) | 49 (17.8) | 3,504 (17.3) |  |
| **Sex** |  |  |  |  |
| Male | 11,683 (57.1) | 182 (65.9) | 11,501 (56.9) |  |
| Female | 8.794 (42.9) | 94 (34.1) | 8,700 (43.1) |  |
| **Duration of follow-up** | 4.49 ± 3.57 | 1.46 ± 1.78 | 4.54 ±3.58 |  |
| **Comorbidities** |  |  |  |  |
| Diabetes mellitus | 5,504 (26.9) | 86 (31.2) | 5,418 (26.8) | 0.11 |
| Hypertension | 6,176 (30.2) | 925 (33.3) | 6,804 (30.1) | 0.25 |
| Asthma | 4,183 (20.4) | 47 (17.0) | 4,136 (20.5) | 0.16 |
| COPD | 1,895 (9.3) | 22 (8.0) | 1,873 (9.3) | 0.46 |
| Liver Cirrhosis | 355 (1.7) | 3 (1.1) | 352 (1.7) | 0.64 |
| Chronic kidney disease | 617 (3.0) | 13 (4.7) | 604 (3.0) | 0.10 |
| Solid cancer | 4,535 (22.1) | 60 (21.7) | 4,475 (22.2) | 0.87 |
| Hematologic malignancy | 12,442 (60.8) | 170 (61.6) | 12,272 (60.7) | 0.78 |
| Autoimmune disease | 222 (1.1) | 1 (0.4) | 222 (1.1) | 0.38 |
| **Charlson comorbidity index** | 5.13 ± 3.09 | 5.15 ± 3.16 | 5.13 ± 3.09 | 0.95 |
| **Risk factors** |  |  |  |  |
| Previous TB History | 422 (2.1) | 11 (4.0) | 411 (2.0) | 0.024 |
| Pulmonary TB | 230 (1.1) | 10 (3.6) | 220 (1.1) | 0.001 |
| Extrapulmonary TB | 250 (1.2) | 2 (0.7) | 248 (1.2) | 0.78 |
| **Underlying disease entities for HSCT** | |  |  |  |
| AML | 4,920 (24.0) | 83 (30.1) | 4,837 (23.9) | 0.018 |
| ALL | 2,846 (13.9) | 41 (14.9) | 2,805 (13.9) | 0.64 |
| CML | 369 (1.8) | 5 (1.8) | 364 (1.8) | >0.99 |
| Lymphoma | 4,315 (21.1) | 45 (16.3) | 4,270 (21.1) | 0.05 |
| Multiple myeloma | 4,155 (20.3) | 42 (15.2) | 4,113 (20.4) | 0.035 |
| Severe aplastic anemia | 8,066 (39.4) | 105 (38.0) | 7,961 (39.4) | 0.65 |
| MDS | 2,131 (10.4) | 51 (18.5) | 2,080 (10.3) | <0.001 |
| Others | 3,975 (19.4) | 49 (17.8) | 3,926 (19.4) | 0.48 |
| **HSCT source** |  |  |  |  |
| BMT | 1,083 (5.3) | 16 (5.8) | 1,067 (5.3) | 0.85 |
| Peripheral blood | 19,013 (92.9) | 256 (92.8) | 18,757 (92.9) |  |
| Umbilical Cord blood | 366 (1.8) | 4 (1.4) | 362 (1.8) |  |
| **Transplant year** |  |  |  |  |
| 2009 | 1,316 (6.4) | 41 (14.9) | 1,275 (6.3) | <0.001 |
| 2010 | 1,439 (7.0) | 41 (14.9) | 1,398 (6.9) |  |
| 2011 | 1,500 (7.3) | 27 (9.8) | 1,473 (7.3) |  |
| 2012 | 1,525 (7.4) | 32 (11.6) | 1,493 (7.4) |  |
| 2013 | 1,568 (7.7) | 21 (7.6) | 1,547 (7.7) |  |
| 2014 | 1,719 (8.4) | 27 (9.8) | 1,692 (8.4) |  |
| 2015 | 1,714 (8.4) | 17 (6.2) | 1,697 (8.4) |  |
| 2016 | 1,837 (9.0) | 19 (6.9) | 1,818 (9.0) |  |
| 2017 | 1,871 (9.1) | 18 (6.5) | 1,853 (9.2) |  |
| 2018 | 1,959 (9.6) | 11 (4.0) | 1,948 (9.6) |  |
| 2019 | 2,163 (10.6) | 16 (5.8) | 2,147 (10.6) |  |
| 2020 | 1,866 (9.1) | 6 (2.2) | 1,860 (9.2) |  |
| **Outcome** |  |  |  |  |
| Death | 6,295 (30.7) | 134 (48.6) | 6,161 (30.5) | <0.001 |

Note: Data are expressed number (percent) or mean ± standard deviation.

Abbreviations: ALL, acute lymphoblastic leukemia; AML, acute myeloid leukemia; BMT, bone marrow transplantation; CML, chronic myelogenous leukemia; COPD, chronic obstructive pulmonary disease; HSCT, hematopoietic stem cell transplantation; MDS, myelodysplastic syndrome; SOT, solid organ transplantation; TB, tuberculosis
