# Supplementary material for: Changes in tuberculosis risk after transplantation in the setting of decreased community tuberculosis incidence: a national population-based study, 2008–2020
Source: Ann Clin Microbiol Antimicrob. 2024 Jan 3;23:1. doi: 10.1186/s12941-023-00661-4 (PMC10765802; doi:10.1186/s12941-023-00661-4)
Supplement: Supplementary file 8 — Additional file 8: Table S8. Pulmonary and extrapulmonary tuberculosis according to transplant type. [file 12941_2023_661_MOESM8_ESM.docx]

**Supplementary Table 8.** **Pulmonary and extrapulmonary tuberculosis according to transplant type**

|  | **Pulmonary TB**  (n = 537) | **Extrapulmonary TB**  **(n = 286)** | ***p*-value** |
| --- | --- | --- | --- |
| SOT |  |  | 0.25 |
| Kidney^*^ | 189 (35.2) | 104 (36.4) | 0.74 |
| Heart | 12 (2.2) | 7 (2.4) | 0.85 |
| Liver | 148 (27.61) | 74 (25.9) | 0.60 |
| Lung | 10 (1.9) | 1 (0.4) | 0.11 |
| Others^†^ | 2 (0.42) | 2 (0.7) | 0.61 |
| HSCT |  |  |  |
| Allogeneic | 113 (21.0) | 74 (25.9) | 0.12 |
| Autologous | 63 (11.7) | 24 (8.4) | 0.14 |

*Kidney includes kidney and kidney-pancreas transplantation. ^†^Others include small bowel transplantation and pancreas transplantation alone.

Abbreviations: HSCT, hematopoietic stem cell transplantation; SOT, solid organ transplantation; TB, tuberculosi
