# Supplementary material for: Changes in tuberculosis risk after transplantation in the setting of decreased community tuberculosis incidence: a national population-based study, 2008–2020
Source: Ann Clin Microbiol Antimicrob. 2024 Jan 3;23:1. doi: 10.1186/s12941-023-00661-4 (PMC10765802; doi:10.1186/s12941-023-00661-4)
Supplement: Supplementary file 9 — Additional file 9: Table S9. Risk factors associated with the development of TB after SOT. [file 12941_2023_661_MOESM9_ESM.docx]

**Supplementary Table 9.** **Risk factors associated with the development of TB after SOT**

|  |  | **Univariate** | |  | **Multivariate** | |
| --- | --- | --- | --- | --- | --- | --- |
|  |  | **HR (95% CI)** | ***p*-value** |  | **HR (95% CI)** | ***p*-value** |
| **Age** | 0 to 19 years | 0.13 (0.03, 0.53) | 0.005 |  | 0.13 (0.03, 0.52) | 0.004 |
|  | 20 to 39 years | 1 (ref.) |  |  | 1 (ref.) |  |
|  | 40 to 59 years | 1.35 (1.04, 1.75) | 0.024 |  | 1.29 (0.99, 1.68) | 0.06 |
|  | over 60 years | 2.04 (1.54, 1.65) | <0.001 |  | 1.93 (1.44, 2.60) | <0.001 |
| **Sex** | M | 1.38 (1.14, 1.65) | <0.001 |  | 1.32 (1.09, 1.59) | 0.004 |
|  | F | 1 (ref.) |  |  | 1 (ref.) |  |
| **Diabetes mellitus** | Yes | 1.24 (1.05, 1.46) | 0.013 |  | 1.11 (0.93, 1.32) | 0.25 |
|  | No | 1 (ref.) |  |  | 1 (ref.) |  |
| **Hypertension** | Yes | 0.95 (0.79, 1.14) | 0.59 |  | 0.92 (0.74, 1.14) | 0.44 |
|  | No | 1 (ref.) |  |  | 1 (ref.) |  |
| **Asthma** | Yes | 1.12 (0.85, 1.46) | 0.42 |  | 1.13 (0.85, 1.50) | 0.40 |
|  | No | 1 (ref.) |  |  | 1 (ref.) |  |
| **COPD** | Yes | 1.06 (0.68, 1.65) | 0.80 |  | 0.840 (0.52, 1.350) | 0.47 |
|  | No | 1 (ref.) |  |  | 1 (ref.) |  |
| **Liver cirrhosis** | Yes | 1.16 (0.98, 1.39) | 0.09 |  | 0.92 (0.67, 1.26) | 0.60 |
|  | No | 1 (ref.) |  |  | 1 (ref.) |  |
| **Chronic kidney disease** | Yes | 1.89 (0.75, 1.05) | 0.16 |  | 0.25 (0.72, 2.15) | 0.43 |
|  | No | 1 (ref.) |  |  | 1 (ref.) |  |
| **TB history** | Yes | 1.39 (0.94, 2.05) | 0.10 |  | 1.34 (0.90, 2.00) | 0.15 |
|  | No | 1 (ref.) |  |  | 1 (ref.) |  |
| **Transplantation** | Liver | 1.16 (1.98, 1.39) | 0.09 |  | 1.39 (0.74, 2.60) | 0.31 |
|  | Kidney^*^ | 1 (ref.) |  |  | 1 (ref.) |  |
|  | Heart | 1.00 (0.63, 1.59) | 1.00 |  | 1.18 (0.63, 2.21) | 0.60 |
|  | Lung | 1.43 (0.78, 2.62) | 0.24 |  | 1.45 (0.63, 3.35) | 0.39 |
|  | Others^†^ | 1.59 (0.60, 4.27) | 0.36 |  | 2.30 (0.76, 6.97) | 0.14 |

*Kidney includes kidney and kidney-pancreas transplantation. ^†^Others include small bowel transplantation and pancreas transplantation alone.

Abbreviations: CI, confidence interval; COPD, chronic obstructive pulmonary disease; HR, hazard ratio; SOT, solid organ transplantation; TB, tuberculosis
