# Supplementary material for: Changes in tuberculosis risk after transplantation in the setting of decreased community tuberculosis incidence: a national population-based study, 2008–2020
Source: Ann Clin Microbiol Antimicrob. 2024 Jan 3;23:1. doi: 10.1186/s12941-023-00661-4 (PMC10765802; doi:10.1186/s12941-023-00661-4)
Supplement: Supplementary file 10 — Additional file 10: Table S10. Risk factors associated with the development of TB after HCST. [file 12941_2023_661_MOESM10_ESM.docx]

**Supplementary Table 10.** **Risk factors associated with the development of TB after HCST**

|  |  | **Univariate** | |  | **Multivariate** | |
| --- | --- | --- | --- | --- | --- | --- |
|  |  | **HR (95% CI)** | ***p*-value** |  | **HR (95% CI)** | ***p*-value** |
| **Age** | 0 to 19 years | 0.515 (0.33, 0.81) | 0.004 |  | 0.55 (0.35, 0.87) | 0.010 |
|  | 20 to 39 years | 1 (ref.) |  |  | 1 (ref.) |  |
|  | 40 to 59 years | 0.99 (0.73, 1.34) | 0.93 |  | 1.10 (0.80, 1.51) | 0.56 |
|  | over 60 years | 0.97 (0.66, 1.42) | 0.88 |  | 1.14 (0.75, 1.73) | 0.55 |
| **Sex** | M | 1.460 (1.14, 1.87) | 0.003 |  | 1.49 (1.16, 1.91) | 0.002 |
|  | F | 1 (ref.) |  |  | 1 (ref.) |  |
| **Diabetes mellitus** | Yes | 1.26 (1.98, 1.63) | 0.07 |  | 1.14 (0.87, 1.50) | 0.34 |
|  | No | 1 (ref.) |  |  | 1 (ref.) |  |
| **Hypertension** | Yes | 1.19 (0.93, 1.53) | 0.17 |  | 1.10 (0.84, 1.44) | 0.48 |
|  | No | 1 (ref.) |  |  | 1 (ref.) |  |
| **Asthma** | Yes | 0.79 (0.58, 1.08) | 0.13 |  | 0.82 (0.60, 1.13) | 0.23 |
|  | No | 1 (ref.) |  |  | 1 (ref.) |  |
| **COPD** | Yes | 0.95 (0.62, 1.47) | 0.82 |  | 0.85 (0.55, 1.33) | 0.48 |
|  | No | 1 (ref.) |  |  | 1 (ref.) |  |
| **Liver cirrhosis** | Yes | 0.64 (0.21, 1.99) | 0.44 |  | 0.55 (0.18, 1.71) | 0.30 |
|  | No | 1 (ref.) |  |  | 1 (ref.) |  |
| **Chronic kidney disease** | Yes | 0.65 (0.95, 2.88) | 0.08 |  | 1.81 (1.01, 3.23) | 0.045 |
|  | No | 1 (ref.) |  |  | 1 (ref.) |  |
| **TB history** | Yes | 2.03 (1.11, 3.71) | 0.02 |  | 2.00 (1.09, 3.69) | 0.03 |
|  | No | 1 (ref.) |  |  | 1 (ref.) |  |
| **Transplantation** | Allogeneic HSCT | 1.77 (1.17, 2.28) | <0.001 |  | 2.07 (1.57, 2.73) | <0.001 |
|  | Autologous HSCT | 1 (ref.) |  |  | 1 (ref.) |  |

Abbreviations: CI, confidence interval; COPD, chronic obstructive pulmonary disease; HR, hazard ratio; HSCT, hematopoietic stem cell transplantation; TB, tuberculosis
